# Targeting HMGB3/hTERT axis for radioresistance in cervical cancer

**DOI:** 10.1186/s13046-020-01737-1

**Published:** 2020-11-13

**Authors:** Zongjuan Li, Yang Zhang, Silei Sui, Yijun Hua, Anshi Zhao, Xiaoyuan Tian, Ruonan Wang, Wei Guo, Wendan Yu, Kun Zou, Wuguo Deng, Liru He, Lijuan Zou

**Affiliations:** 1 The Second Affiliated Hospital & Institute of Cancer Stem Cell, Dalian Medical University, Dalian, China; 2 Qingdao University Medical College Affiliated Yantai Yuhuangding Hospital, Yantai, China; 3 SunYat-sen University Cancer Center; State Key Laboratory of Oncology in South China; Collaborative Innovation Center of Cancer Medicine, Guangzhou, China; 4 The First Affiliated Hospital, Dalian Medical University, Dalian, China

**Keywords:** Cervical cancer, HMGB3, hTERT, Radioresistance

## Abstract

**Background:**

Radiotherapy is regarded as a milestone for the cure of cervical cancer. However, clinical outcome heavily be hindered by radioresistance. So, exploring the underlying mechanism of radioresistance, and find potential target, well deserve fully emphasis.

**Methods:**

In this study, we developed two novel radiation resistance cervical cancer cell lines, which could mimic clinical radioresistance. In order to find new potential targets, RNA-Seq, database analysis, streptavidin-agarose and LC/MS were used. Pull-down, luciferase and rescue assays were conducted to explore the regulatory mechanisms. To further evaluate the correlation between therapeutic responses and HMGB3/hTERT expression, 172 cervical cancer patients were recruited.

**Results:**

Knockdown of HMGB3 significantly inhibit the DNA damage repair and induced more γH2AX foci, leading to enhanced chemo- and radio-sensitivity in vitro and in vivo, whereas HMGB3 overexpression has the opposite effects. HMGB3 promotes cell growth and radioresistance by transcriptionally up-regulating hTERT via the specifical binding of HMGB3 at the hTERT promoter region from − 902 to − 321. HMGB3 knockdown-mediated radiosensitization could be reversed by the overexpressed hTERT in both cervical cancer cell lines and xenograft tumor mouse model. Furthermore, clinical data from 172 cervical cancer patients proved that there was a positive correlation between HMGB3 and hTERT expression, and high expression of HMGB3/hTERT predicted poor response to radiotherapy, worse TNM stages and shorter survival time.

**Conclusion:**

Here, we have identified HMGB3/hTERT signaling axis as a new target for cervical cancer radioresistance. Our results provide new insights into the mechanism of cervical cancer radioresistance and indicate that targeting the HMGB3/hTERT signaling axis may benefit cervical cancer patients.

**Supplementary information:**

**Supplementary information** accompanies this paper at 10.1186/s13046-020-01737-1.

## Background

Cervical cancer is one of the leading causes of cancer related-death in women worldwide [1, 2]. Radiotherapy, alone or in combination with chemotherapy, is the mainstream for the cure of cervical cancer [3–5]. Nearly 80% cervical cancer patients received radiation therapy in the process of treatment. However, overall survival was still heavily hampered by radioresistance [6–8]. Thus, discovering and identifying the novel targets of tumorigenesis and radioresistance is of importance for cervical cancer treatment.

Human telomerase reverse transcriptase (hTERT) is a catalytic subunit of telomerase whichcan maintain telomeric integrity. hTERT is activated in most cancer cells but not in most normal cells [9]. As a repair response to radiation-induced DNA damage, an increased activity of hTERT was found in human colon carcinoma, lymphoma, and myeloma cells [10–13]. Previous studies have also found that hTERT could accelerate the process of EMT and stemness, which then lead to treatment resistance, metastasis, and recurrence [14]. hTERT is regulated at multiple molecular levels, among which, transcriptional modulation is the most important [15]. Identification of novel cellular factors, which modulate hTERT in transcriptional process, deserves comprehensive research. In this study, we have first identified high-mobility group box 3 (HMGB3) as a new regulator which specifically bind to hTERT promoter and transcriptionally activate hTERT expression in cervical cancer radioresistant cells.

The high-mobility group (HMG) super family is the most abundant nonhistone proteins in the eukaryotic nucleus [16]. HMG-Box family plays a significant role in DNA replication, transcription, recombination and repair. HMGB1 and 2 can bind to DNA without sequence specificity, and promote the formation of nucleoprotein complex. They can also directly interact with DNA-binding proteins and then affect transcription. HMGB3 has 80% identity with HMGB 1, 2 and may share some functions with them [17]. Previous studies revealed that HMGB3could promote tumor development and maintain dedifferentiation in urinary bladder cancer, oesophageal squamous cancer, gastric cancer, non-small cell lung cancer, breast cancer and hematopathy [18–21]. Nevertheless, there has not yet been a report about the biological function of HMGB3 in regulating cervical cancer radioresistance.

In this study, we examined the regulation of HMGB3 on cervical cancer cell proliferation and apoptosisafter exposing to radiation, and also explored its underlying mechanism including of DNA damage pathways. Moreover, we demonstrated that HMGB3 induced radioresistance by transcriptionally activating hTERT. Finally, we also analyzed the relationship between HMGB3/hTERT signalling axis and clinical outcome in the cervical cancer patients. Our findings demonstrate that targeting the HMGB3/hTERT axis may be a potential promising for the treatment of cervical cancer.

## Methods

### Patients and tissue samples

This study was approved by Ethics Committee of The Second Affiliated Hospital of Dalian Medical University. Fifty three patients diagnosed with cervical squamous cell carcinoma were recruited. Before the end of the statistics, all the patients did not undergo surgical resection, and all the patients received standard concurrent chemoradiotherapy. All patients signed informed consents before being enrolled into research and we comply with the Declaration of Helsinki when conducting the study. Cervical cancer tissue microarrays containing 119 patients were purchased from OutdoBiotech (Shanghai, China). The tumor tissue samples (tumor and adjacent tissue) were took from patients who had not undergone anti-tumor therapy since diagnosis, with all of the information frompatients authenticated.

### Cell lines and cell culture

The human cervical cancer cell lines HeLa, SiHa, C33A, DoTc2, HeLa S3, and CaSki were obtained from American Type Culture Collection (ATCC, VA). HeLa, SiHa, and C33A cells were specifically cultured in Eagle’s minimal essential medium (EMEM) containing 10% foetal bovine serum (FBS), and DoTc2, HeLa S3, and CaSki cells were cultured in Dulbecco’s modified Eagle’s medium (DMEM) containing 10% FBS. All the cells were cultured in a humidified atmosphere with 5% CO2 at 37 °C.

The following human cervical cancer cell lines were plated in 6 cm dishes: HeLa, SiHa, C33A, DoTc2, HeLa S3, and CaSki. After 24 h, the cells reached exponential growth phase (75% density, 1 × 10^6^ cells) and were then exposed to 15 sequential of 2 Gy/day for a total dose of 30 Gy. The subclones from the survival populations display distinct resistance to radiotherapy (here named as RR SiHa and RR HeLa) (there were no survival in the other four cervical cancer cell lines after 30 Gy radiation).

### Antibodies

Anti-hTERT was purchased from Millipore. Caspase-3, Caspase-9, PARP and γH2AX antibodies were purchased from Cell Signaling Technology (MA, USA). GAPDH, β-actin, HMGB3 and antibodies were purchased from Proteintech (Wuhan, China). H3K4me3 antibody was purchased from Absin Bioscience (China).

### Clonogenic survival assay

When reached exponential growth phase, the cervical cancer cell were trypsinized, counted and seeded different numbers into 60 mm culture dishes according to different radiation doses: 0 Gy (200 cells), 2 Gy (500 cells), 4 Gy (500 cells), 6 Gy (1000 cells), 8 Gy (2000 cells),10 Gy (2000 cells). After 24 h incubation, the cells adhere to the dishes and were performed radiation treatment. For 10–14 days at 37 °C with 5% CO2, the cells were fixed with methanol: glacial: acetic(1:1:8) for 10 min, and stained with 0.1% crystal violet for 30 min. Calculate the clone formation rates and survival fractions, and the obtained values were analyzed by Prism 8 software to fit the single-hit multi-target model (Y = 1-(1-exp(−n*X))^m).

### Comet assay

Briefly, cells were harvested following the indicated treatments and were mixed with low-melting-point agarose. The samples were then immersed into the fresh lysis buffer (10 mM Tris-HCl, 2.5 M NaCl, 100 mM EDTA, 1% Triton X-100, and 10% DMSO) for 1 h at 4 °C and neutralized for 15 min. Then the slides were subjected to electrophoresis at 21 V for 20 min under alkaline conditions. Slides were stained with ethidium bromide solution (20 μg/ml) and photoed using a fluorescence microscope (Leica DMI4000B). We analysed 100 individual cells from each group by Comet Assay Software (CaspLab). The tail moment served as a quantitative measure of DNA damage..

### High-throughput mRNA-sequence and data analysis

Cells were harvested following the indicated treatments and resuspended into RIzolreagent(Invitrogen). RNA sample quantification, qualification, library preparation and subsequent RNA sequencing were performed by Novogene Co., LTD (Beijing, China). EdgeR R package (3.12.1) was used to analyse the differential expression of the samples. Corrected *P*-values of 0.05 and absolute fold-changes of 2 were set as the threshold for significantly different expression.

### siRNA design and transfection

The HMGB3-specific siRNAs (siRNA1, F 5′-GGAAGUGAUCAUCUCCGAUTT-3′, R 5′-AUCGGAGAUGAUCACUUCCTT-3′; siRNA2, F 5′-GGUCUUCGCCUUGAUUCAUTT-3′, R 5′- AUGAAUAAGGCGAAGACCTT-3′) and negative control siRNA were purchased from GenePharma (Shanghai, China). Transient transfection was performed by using Lipofectamine 2000 (Invitrogen, Carlsbad, CA, USA) according to the manufacturers’ protocols. Briefly, cervical cancer cells were plated into 96-well plates or six-well plates. The cells were transfected with siRNA 1, siRNA 2, or negative control (1–2 μg) for 6–8 h and then replaced with the fresh medium. After 12 h, the cells were exposed to radiation. At 24 h, the cells were harvested for different analysis.

### Plasmid vectors and transfection

HMGB3-targeting shRNAs (sh1, sh2, sh3) and negtive control shRNA plasmids were purchased from GeneCopoeia. The lentivirus mediated vectors were transfected using Lenti-Pac HIV expression packaging kit according to the manufacturer’s instructions (GeneCopoeia). Briefly, 1.3–1.5 × 10 ^6^ 293 T cells were plated into one 10 cm culture dish and were transfected with 2.5 μg shRNA expression plasmids and 5.0μllenti-Pac HIV mix usingLipofectamine 3000 (Invitrogen). After 7-8 h, the mix was replaced with fresh complete medium. The viral supernatants were collected after 48 h and used for lentiviral infection obtaining stably transduced cells.

### MTT assay

The cells were seeded in 96-well plates (2000 cells/well) and then transfected with HMGB3 specific siRNAs. After 24 h, the cells were treated with different dose of DDP, Vin and TAX. Forty-eight hours later, add MTT reagent and incubate for 3 h. Then, MTT was replaced with 150 μl of dimethyl sulfoxide (DMSO). The OD value was measured at 490 nm.

### Immunofluorescence

Cells were planted in six well plate with coverslips and treatment as indicated. The coverslips were fixed and were then immersed into 0.2% Triton X-100 for 3 min. 10% bovine serum albumin was used for 30 min to eliminate the nonspecific binding. The cells were then incubated with the primary antibodies (1:200) overnight at 4 °C. After washing with PBS, the cells were incubated with corresponding secondary antibody labelled with fluoresce for 1 h at room temperature. The nucleus was stained with DAPI. The immunofluorescence was analysed by confocal laser scanning microscope (Leica).

### Dual luciferase assay

The promoter of hTERT (− 1655 to + 40) was cut into 4 segments and each segment was inserted into a luciferase reporter vector pGL3. The cervical cancer cells were seeded into a 6-well plate and transfected with HMGB3 siRNA or a negative control for 6–7 h. At 24 h, the cells were transfected with the hTERT reporter or a pRL-TK control plasmid. After 48 h, the luciferase activity was measured according to the kit Dual-Luciferase® Reporter Assay System (Promega, Cat: E1910).

### Chromatin immunoprecipitation assay

ChIP assays were performed as previously described [9]. Firstly, the cells were treatment with indication and then fixed with 1% formaldehyde for 15 min at room temperature. 10% 1.25 M glycine was then added into for 5 min to prevent excessive cross linking. Scraped, collected, and sonicated the samples on ice to cleave the DNA into 100–1000 bp fragments. Then, part of the cell lysate was incubated with protein A/G agarose beads (Santa Cruz Biotechnology) and HMGB3 or IgG antibody. Part of the cell lysate was used for the DNA input. The mixture was washed and reverse cross-linked at 65 °C for 12 h. The DNA was extracted by phenol/chloroform and used as a template to PCR. The primers: F 5′- TTTCCCACCCTTTCTCGACG-3′; and R 5′-CAGCGGAGAGAGGTCGAATC − 3′.

### Pulldown assay

The biotin-labelled hTERT promoter probe corresponding to − 1645 to + 40 was synthesized by Sigma (Sigma, USA) (F 5′-GACACACTAACTGCACCCAT-3′ and R 5′-ACGCAGCGCTGCCTGAAACT-3′). Nucleoprotein extract (400 μg) mixed with 4 μg of hTERT promoter probe and 45 μl of streptavidin-agarose beads (Sigma, USA) and incubated at room temperature to pull down the DNA-protein complexes. After 2 h, the complex was collected by centrifugation, and boiled at 100 °C for further analysis.

### Xenograft models studies

Animal study has been approved by Animal Care and Ethics Committee of Dalian Medical University. Female nude mice (Balb/c) were obtained from Beijing Vital River Laboratory Animal Technology (Beijing, China). Mice were randomly grouped. Cervical cancer cells were implanted subcutaneously in left armpit. The mice were placed on the plate after anesthesia and was irradiated as the “Irradiation” described. After 2 weeks, mice were sacrificed, and tumours were removed. The tumour tissue was stored with 4% formaldehyde or liquid nitrogen for further analysis.

### Immunohistochemical staining

The tissue was immersed in paraffin and sliced. The mice tissue slides and human cervical cancer tissue microarray was processed as follows: dewaxing, rehydration, antigen repair, blocking. After that, the slides were incubated with the primary antibodies overnight, incubated with the HRP-labeled second antibody, developed with DAB and then counterstained with hematoxylin. Staining intensity was defined as: 0 points are (−), 2–3 points are(±), 4–5 points are (+), and 6–7 points are (++). In the final evaluation,(+) (++) was judged to be positive.

### Irradiation

Irradiation of cell lines was performedby an X-RAD320 instrument using an X-ray generator (dose rate: 0.40 Gy/min, target distance: 50 cm, Precision X-Ray Inc., North Branford, CT, USA).Animal irradiations were performed with6MV X-ray (2 Gy/day, a rate of 1.32 Gy/min with 320 keV (peak), 6 mA, filtered with 2 mm Al, radiated every 2 days, 12Gy totally).

### Statistical analysis

Each experiment was repeated 3 times under the same conditions. Student’s t tests were used to compare the continuous variables in both test and control groups. Cumulative survival probability was calculated by Kaplan-Meier analysis. Cox-regression model was performed for multivariate analysis, hazard radios (HRs) and 95% confidence intervals (CIs). All the data in the research were analysed using SPSS 20 software (Inc., Chicago, IL). A 2-sided *p*-value 0.05 was defined to be significant.

## Results

### Cervical cancer RR SiHa and RR HeLa cells possesses radioresistant abilities

**Fig. 1:**
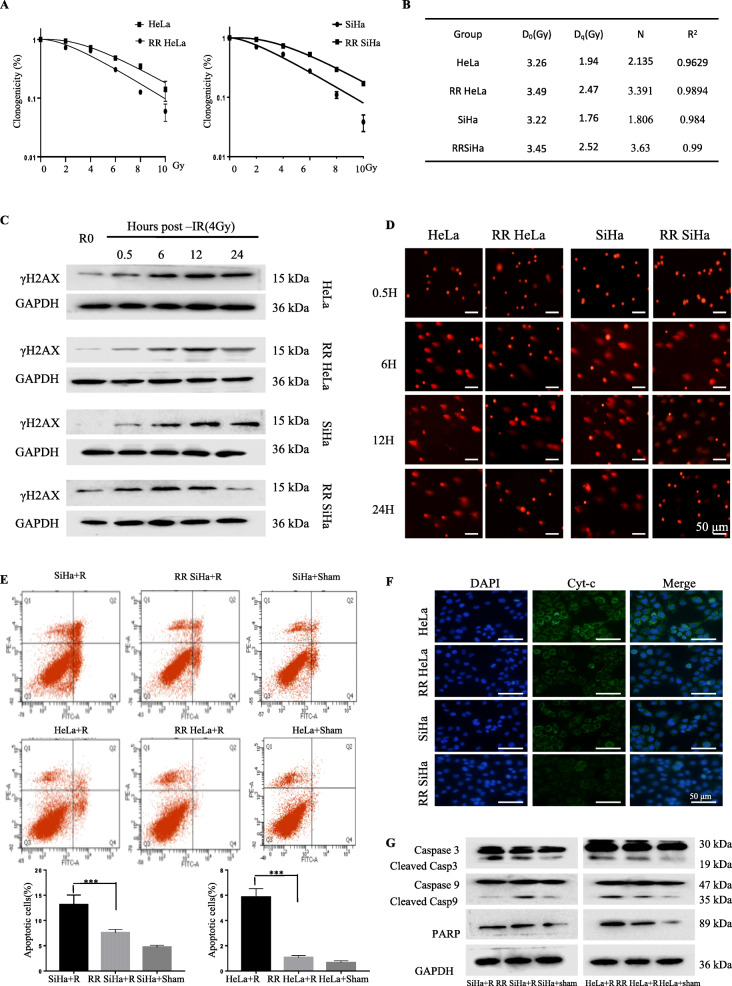
Cervical cancer RR Siha and RR Hela cells possesses radioresistant abilities. **a** The ability of colony formation in the radioresistant and parallel cervical cancer cells after radiation. **b** Biological parameters of radioresistant and parallel cervical cancer cells after radiation. **c** Immunoblotting of γH2AX at different time point in SiHa, HeLa and RR SiHa, RR HeLa cells after treated with 4 Gy radiation. **d** Comet assay was performed to detect DNA damage after exposed to 4 Gy radiation in HeLa, RR HeLa, SiHa and RR SiHa cells at the different time point. **e** 24 h after 4Gy radiation, apoptosis in the cells was detected by FACS analysis. **f** HeLa, SiHa, RR HeLa and RR SiHa were exposed to 4 Gy radiation. 24 h later, the cytochrome-c release was detected by immunofluorescence assay. **g** The expression level of caspase-3,caspase-9 and cleaved-PARP were detected by western blot

Six human cervical cancer cell lines HeLa, SiHa, C33A, DoTc2, HeLa S3, and CaSki (1 × 10^6^) were exposed to a total dose of 30 Gy/15 fractions. Only HeLa and SiHa cells survived after 30 Gy irradiation (data not shown). The subclones from the survival populations were named asRR HeLa and RR SiHa. To confirm the radioresistance of RR HeLa and RR SiHa, cells were exposed to a series of single radiation dose (0-10Gy) and the clonogenicity were examined subsequently. The results showed there was an increase in D0, Dq, N and survival rates in RR HeLa and RR SiHa, indicating a reduction in the radiosensitivity (Fig. 1a, b).We also verified the radioresistance of RR HeLaand RR SiHaby analyzing the pro-survival proteins, and showed that the phosphorylation levels of p110α, p110β, and AKT, which are the hallmarkers of cancer radioresistance, were markedly increased in the RR HeLa and RR SiHa cells (Additional file 1: Figure S1A).

Radiation causes DSBs, which then induce the formation of γH2AX foci. The presence of γH2AX foci indicates the delay of DNA repair, and correlates with radiosensitivity [22]. As shown in Fig. 1c, γH2AX was significantly up-regulated after radiation, but decreased at 24 h after exposure to radiation treatment in RR HeLa and RR SiHa cells, indicating that radioresistant cells could repair DNA lesions more effectively. In addition, comet assay was also used to verify the damaged DNA. The results showed that, within 12 h of receiving radiation, the comet tails were significantly longer. But, at 24 h after radiation treatment, the average tail intensity (percentage of DNA in the tail) in the RR HeLa and RR SiHa cells was attenuated compared with the parental cells (Fig. 1d, Additional file 1: Figure S1B). The reduced γH2AX was also observed in the RR HeLa and RR SiHa cells by immunofluorescence assays (Additional file 1: Figure S1C).

Dysregulated DNA damage diminishes the susceptibility of the irradiated cells to cell death [23]. We next detected the apoptosis of radioresistant cervical cancer cells after receiving radiation. Consistently, RR HeLa and RR SiHa cells abrogated apoptosis after radiation (Fig. 1e), resulting in the less release of cytochrome-c from the inter mitochondrial space into the cytosol (Fig. 1f) and the lower expression of cleaved-PARP, cleaved-caspase-3 and cleaved-caspase-9 (Fig. 1g). These results further confirmed RR HeLa and RR SiHa cells possessed radioresistant abilities.

### HMGB3 binds to hTERT promoter and activates hTERT transcription in cervical cancer radioresistant cells

**Fig. 2:**
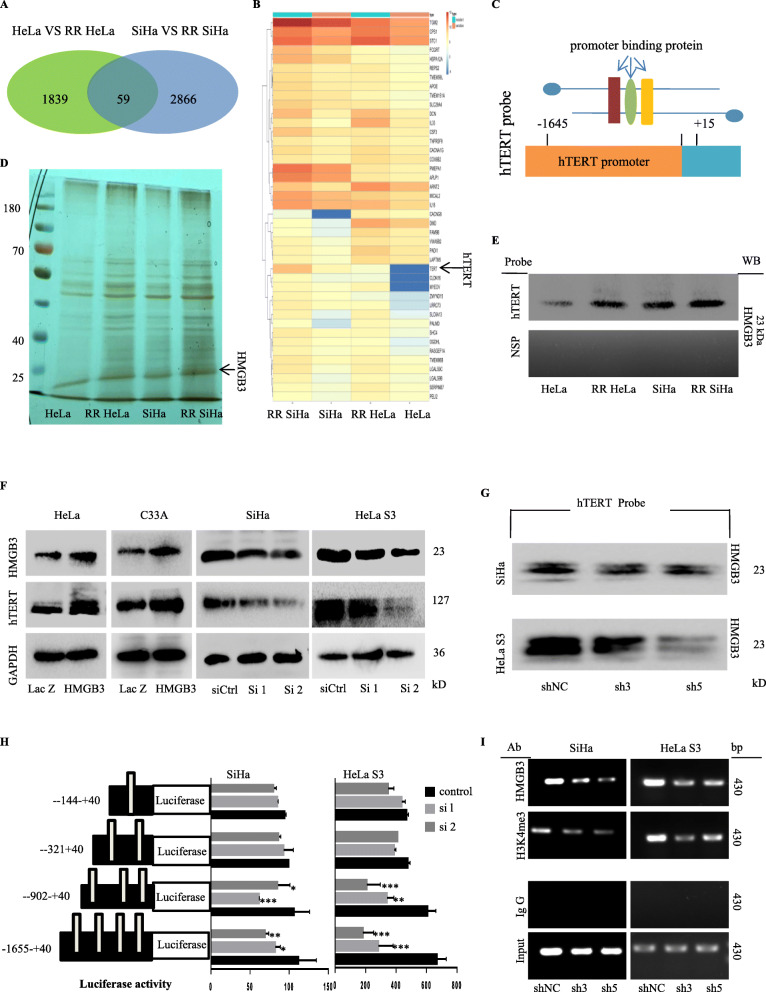
HMGB3was identified as a new transcriptional factor of hTERT in cervical cancer radioresistant cells. **a** Differential gene expression between radioresistant and parental cervical cancer cells from RNA-Seq. **b** A heatmap showing 42 significantly up-regulated genes in the RR SiHa and RR HeLa. **c** A demonstration map of hTERT promoter probe with a biotin from − 1645 to + 15. **d** HMGB3 was identified as a new transcriptional factor of hTERT by streptavidin-agarose and LC/MS. **e** Biotin-streptavidin pulldown was perform to further verify the binding of HMGB3 on the hTERT promoter. **f** Cervical cancer cells were transfected with HMGB3-overexpressing plasmids or HMGB3 specific siRNAs. The expression of HMGB3 and hTERT were detected by western blot. **g** SiHa was transfected with HMGB3 specific siRNAs, and then the binding ability of HMGB3 on hTERT promoter (− 1645 to + 15) was detected by pulldown assay. **h** SiHa and HeLa S3 cells were transfected with HMGB3 specific siRNAs, and the activity of different fragments of hTERT promoter was detected by Luciferase Reporter assay. **i** CHIP assay was used to verify the binding of HMGB3 and H3K4me3 on region − 902 to − 321 of hTERT promoter

To identify the underlying mechanism of radioresistance in cervical cancer, we examined the expression of transcriptomes in radioresistant and parental cervical cancer cell lines by RNA-seq analysis. A total of 59 differentially expressed genes were identified (DEGs, upregulated: 42; downregulated: 17) (Fig. 2a) in radioresistant cells. Heat map analysis showed that hTERT expression was comparatively high in radioresistant cervical cancer cell lines (Fig. 2b). Western blot assay furtherly verified the upregulation of hTERT in these radioresistant cells (Additional file 1: Figure S2A). Previous researches have shown that irradiation and chemotherapy can modulate the expression of the hTERT in vitro and in vivo [13], and the activation of hTERT was recognized as a repair response to radiation-induced DNA damage [11]. Accordingly, we are of great interest to know why hTERT is highly activated in the cervical cancer radioresistant cells and whether hTERT plays a vital role in cervical cancer radioresistance and progression.

Next, we detected and identified the tumor-specific cellular factors which bound to the hTERT promoter and regulated hTERT expression and cervical cancer radioresistance by streptavidin-agarose pulldown assay. The nuclear extracts were incubated with streptavidin-agarose beads and a biotin-labelled hTERT promoter probe corresponding − 1645 to + 15 (Fig. 2c, Additional file 1: Figure S2B). Then, the candidates were pulled down and analyzed by the mass spectrometry and proteomic techniques. A protein band (~ 25 kDa) with high binding activity at the hTERT promoter was identified as HMGB3 in RR cervical cancer cell lines (Fig. 2d). Immunoblot assay confirmed the stronger binding ability of HMGB3 on the hTERT promoter in the radioresistant cells (Fig. 2e). We also showed that hTERT and HMGB3 possessed the same subcellular colocalization, and were highly expressed in the cervical cancer radioresistant cells (Additional file 1: Figure S2C, 2D).

HMG has been shown to regulate gene transcription and expression by binding to DNA sequence [24, 25]. We hypothesized that the regulation of HMGB3 on cervical cancer radioresistance was mediated by activation of hTERT transcription. Considering that HMGB3 expression was comparatively lower in HeLa and C33A cells and higher in SiHa and HeLa S3 cells (Additional file 1: Figure S2E), HeLa and C33A cells were chosen for overexpression experiments, SiHa and HeLaS3 cells for knockdown experiments. Firstly, HMGB3 knockdown suppressed hTERT expression, while its overexpression upregulated hTERT expression in cervical cancer cells (Fig. 2f). HMGB3 knockdown also abrogated the binding of HMGB3 at the hTERT promoter (Fig. 2g). To further identify the binding sites of HMGB3 on the hTERT promoter, we constructed the hTERT promoter-driven luciferase reporter vectors with four different regions. The results shown that HMGB3 knockdown significantly inhibited the activity of hTERT promoter corresponding to the fragments from − 1655 to + 40 and from − 902 to + 40, but not from − 321 to + 40 and from − 144 to + 40 (Fig. 2h). These results demonstrated that HMGB3 bound to the hTERT promoter region from − 902 to − 321. ChIP assay also verified the binding at this region (Fig. 2i). HMGB3 knockdown also inhibited the binding of H3K4me3, a chromatin activation marker, at the hTERT promoter in cervical cancer cells (Fig. 2i).

### HMGB3 regulated radio- and chemosensitivity by targeting DNA damage repair and apoptosis pathways in cervical cancer cells

**Fig. 3:**
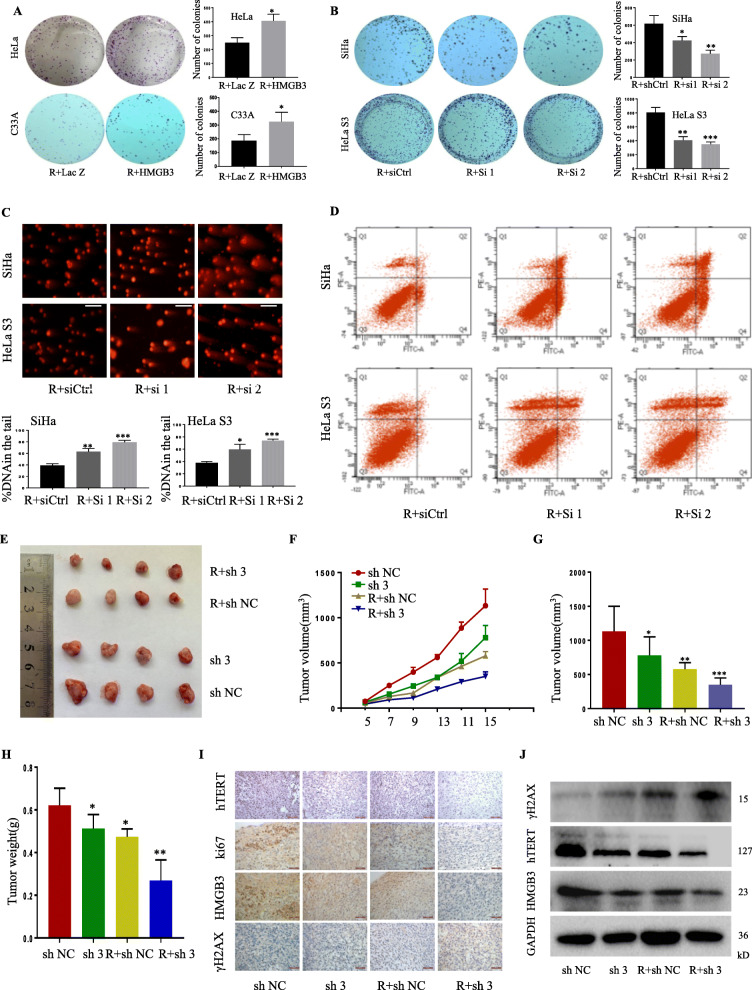
Knockdown of HMGB3 increased the susceptibility to radiotherapy in cervical cancer cells and the xenograft mouse model Cervical cancer cells were transfected with HMGB3-overexpressing plasmids (**a**) or HMGB3 specific siRNAs (**b**) and then exposed to 4Gy radiation. At 24 h, colony formation assay was performed.SiHa and HeLa S3 were transfected with HMGB3 specific siRNAs and then exposed to 4Gy radiation. At 24 h, comet assay was performed to detect DNA damage (**c**), and FACS analysis was performed to detect cell apoptosis(**d**). **e** SiHa was infected by lentivirus-HMGB3 shRNA. Cells were implanted into left armpit of the nude mice. Mice were sacrificed on day 15 and tumors were removed and photographed. **f** Recorded the tumor volume every two days during the course of the experiment.Tumor volume (**g**) and and weight (**h**) was measured and recorded. **i** Immunohistochemical staining for HMGB3, ki67, hTERT and γH2AX. **j**The expression of HMGB3, hTERT and γH2AX proteins in mice tumor tissues were detected by Western blot

We furtherly study the effect of HMGB3 on radiosensitivity of cervical cancer. Firstly, we found that HMGB3 overexpression increased clonogenic survival upon irradiation compared with the control group (Fig. 3a). By contrast, its knockdown obviously inhibited clonogenic survival upon irradiation (Fig. 3b).Comet assay of DNA damage showed that the tail intensity in the HMGB3-knockdown groups was significantly greater than the control group when exposed to same dose radiation (Fig. 3c). In addition, a stronger γH2AX foci still remained at 24 h after irradiation in the HMGB3-knockdown groups (Additional file 1: Figure S3A). Furthermore, HMGB3 knockdown significantly increased apoptosis after radiation (Fig. 3d, Additional file 1: Figure S3B) and up-regulated the expression of the pro-apoptotic proteins cleaved caspase-3, 9 and cleaved PARP (Additional file 1: Figure S3C). KEGG pathway analysis indicated that cervical cancer radiosensitivity may be related to the PI3K/AKT signal pathway (Additional file 1: Figure S3D). Thus, we next detected the influence of HMGB3 on the PI3K/AKT signaling pathway. The results shown that knockdown of HMGB3 decreased the phosphalyated level of p110α, p110β, AKT, and p85, and upregulated the phosphorylated form of PTEN. Overexpression of HMGB3 showed the opposite results (Additional file 1: Figure S3E).

Chemotherapy is also very important for cervical cancertreatment [26, 27]. We next determined the effect of HMGB3 on sensitivity of cervical cancer cells to three common chemotherapeutic drugs. HMGB3 knockdown markedly increased the susceptibility of the cervical cancer cells to cisplatin (DDP), paclitaxel (TAX) and vincristine (VIN) treatment (Additional file 1: Figure S4A), resulting in a significant reduction in IC50 value (Additional file 1: Figure S4B) and a reduced colony formation (Additional file 1: Figure S4C).

### Knockdown of HMGB3 increased the susceptibility to radiotherapy in the xenograft mouse model

We next validate the effect of HMGB3 on radiosensitivity in mice model. HMGB3 was stably knockdown in SiHa cells, and then injected into nude mice (Additional file 1: Figure S5A, 5B). As shown, HMGB3 knockdown effectively suppressed the tumor growth in response to radiotherapy (Fig. 3e-h). In addition, IHC analysis (Fig. 3i) and immunoblot (Fig. 3j) also demonstrated that HMGB3 knockdown regulated the expression of the tumor-associated proteins in xenografts, resulting in the inhibitions of hTERT and Ki67, and the upregulation of γH2AX.

### The HMGB3-mediated radioresistance depended on its transcriptional regulation of hTERT in cervical cancer

**Fig. 4:**
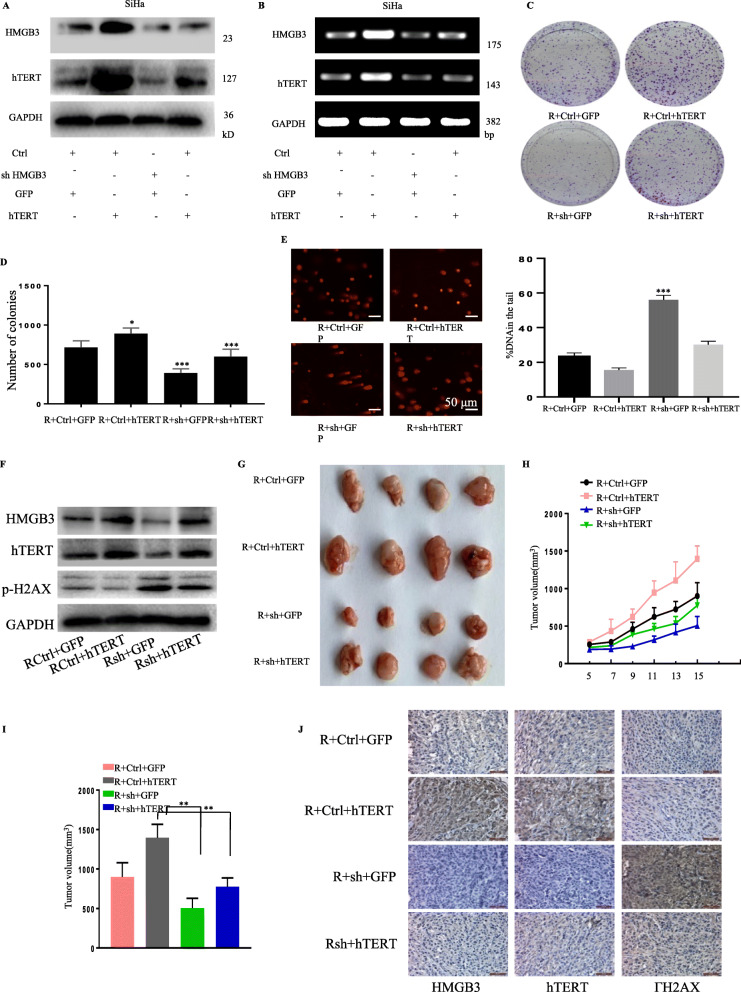
HMGB3 promoted cervical cancer radioresistance by activating hTERT. SiHa was stably silenced of HMGB3 followed by hTERT overexpression. The level of HMGB3 and hTERT were checked by western blot (**a**) and PCR (**b**). **c** SiHa was stably silenced of HMGB3 followed by hTERT overexpression. The colony formation were detected after 4 Gy radiation. **d** Quantification of C. SiHa was stably silenced of HMGB3 followed by hTERT overexpression. The cells were then exposed to 4 Gy radiation. Comet assay was performed after 24 h (**e**). The expression level of γH2AXwas checked by western blot (**f**). Stable SiHa was left armpit of the nude mice. Mice were sacrificed on day 15, and tumors were removed and photographed (**g**).Recorded the tumor volume every two days during the course of the experiment (**h**). Tumor volume was measured and recorded (**i**). **j** Immunohistochemical staining for HMGB3, hTERT and γH2AX in the mice tumor tissue

To validate that the HMGB3-mediated cervical cancer radioresistance depends on its regulation on hTERT, we next performed the rescue experiments in cervical cancer cells and mouse model. In SiHa cells, hTERT was overexpressed after HMGB3 knockdown (Fig. 4a, b). Colony formation assay indicated that hTERT overexpression reversed the HMGB3 knockdown-mediated radiosensitization (Fig. 4c,d). Moreover, hTERT overexpression also reversed the HMGB3 knockdown-mediated inhibitions of DNA damage repair, indicated by degraded comet tail and γH2AX foci (Fig. 4e, f). The results from xenograft mouse model (Fig. 4g-j) further supported that the HMGB3-mediated radioresistance depended on its transcriptional regulation of hTERT in cervical cancer.

### The cervical cancer patients with high hTERT/HMGB3 expression had poor clinical response to radiotherapy

**Fig. 5:**
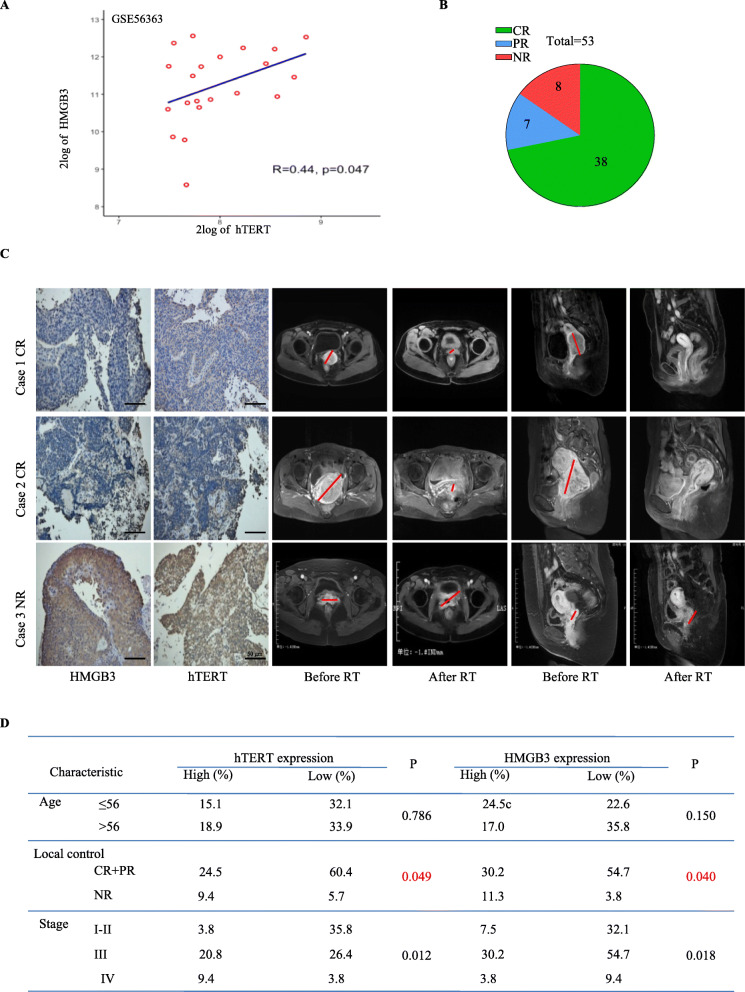
HMGB3 expression was positively correlated with hTERT and high expression of HMGB3/hTERT predicted radioresistancein the cervical patients. **a** Correlation analysis between HMGB3 and hTERT in a GEO database (GSE56363). **b** 53 cervical cancer patients undergoing radiotherapy was recruited. Six mouth after radiothetapy, 38 patients got complete response (CR), 7 patients got partial response (PR), and 8 patients got no reponse (NR) or progressive disease (PD) radiotherapy. **c** The HMGB3/hTERT expression and MRI images in 2 CR and 1 NR cervical cancer patients. **d** Correlation between the expression of HMGB3/hTERT and the clinical characteristics of patients received radiotherapy

We next evaluated the correlation between HMGB3/hTERT expression and radiosensitivity in the cervical patients. Firstly, the published gene expression profile of cervical cancer (GSE56363) indicated a positive correlation between hTERT and HMGB3 in the cervical cancer patients undergoing radiotherapy (*R* = 0.44, *P* = 0.047) (Fig. 5a). We then recruited 53 cervical cancer patients undergoing radiotherapy to evaluate the correlation between therapeutic responses and HMGB3/hTERT expression. All the patients received standard concurrent chemoradiotherapy. Among these patients, 38 patients exhibited a complete response (CR) to radiotherapy, 7 patients exhibited a partial response (PR), and 8 patients exhibited no response (NR) or progressive disease (PD) at 6 months (Fig. 5b). Compared with the MRI data before and after radiotherapy, the tumor volume of the patients with high expression of HMGB3 and hTERT did not significantly change, and the tumor volume of the patients with low expression of HMGB3 and hTERT decreased significantly or even disappeared (Fig. 5c). All the results shown that the patients with high hTERT/HMGB3 expression had poor clinical response to radiotherapy (Fig. 5d).

### HMGB3 expression was positively correlated with hTERT in tumor tissues and overexpression of HMGB3/hTERT predicted poor clinical outcomes in cervical cancer

**Fig. 6:**
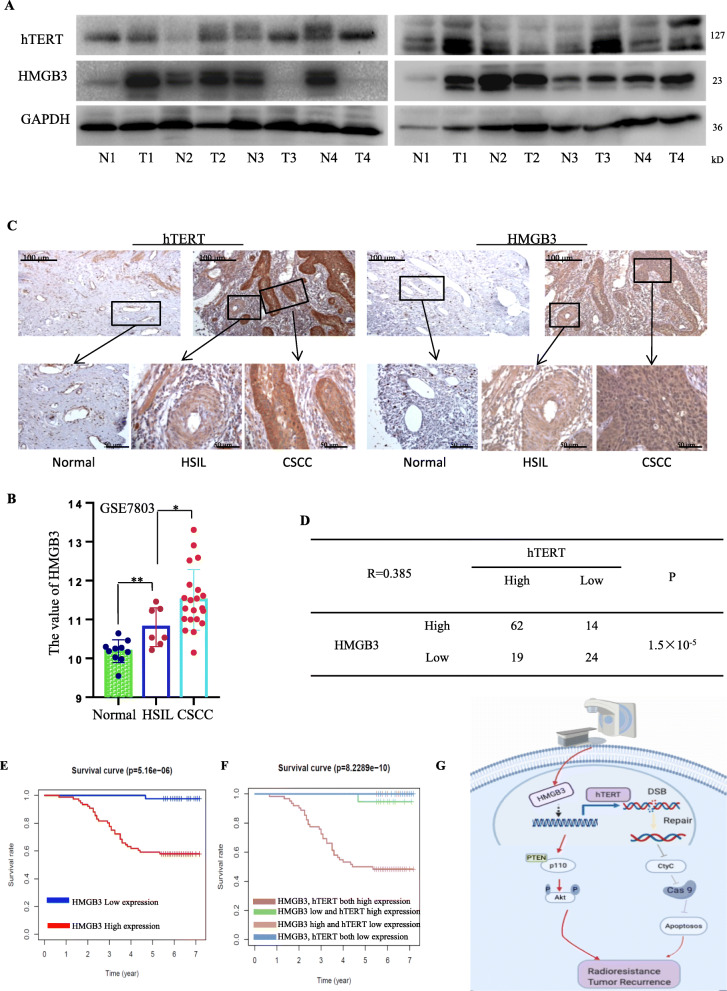
High expression of HMGB3/hTERT predicted poor prognosis in patients with cervical cancer. **a** The expression of HMGB3 and hTERT were detected in the cervical cancer tissue and corresponding adjacent of 8 patients by western blot. **b** HMGB3/hTERT expression analysis form GEO database (GSE7803). A tissue microarray containing 119 cervical cancer patients was used for further study. The tumor tissue samples (tumor and adjacent tissue) were took from patients who had not undergone anti-tumor therapy since diagnosis, with all of the information from patients authenticated. **c** The level of HMGB3 and hTERT in the normal cervical epithelium, high-grade squamous intraepithelial lesion (HSIL) and cervical squamous cell carcinoma (CSCC) (cervical cancer tissue microarrays) were detected by immunohistochemistry. **d** Cox-regression analyses in a tissue microarray containing 119 cervical cancer patients. **e** Survival of the patients with different HMGB3 expression was analyzed by Kaplan–Meier analysis. **f** Overall survival of 119 cervical cancer patients with different HMGB3 and hTERT expression. **g**The schematic diagram of the mechanism ofHMGB3/ hTERT axis in cervical cancer radioresistance

We further confirmed the role of HMGB3/hTERT in the prediction of clinical prognosis. Firstly, we found HMGB3 and hTERT were highly expressed in tumor tissues but moderately expressed in the HSIL compared to the normal cervical epithelium (Fig. 6a-c). A positive correlation (*R* = 0.385, *P* = 1.5 × 10^− 5^) between HMGB3 and hTERT was observed in 119 cervical cancer patients from tissue microarray (Fig. 6d). Among them, 52.1% (62 of 119) patients exhibited both high expression of HMGB3 and hTERT, while 20.2% (24 of 119) exhibited both low expression of HMGB3 and hTERT (Fig. 6d). These results revealed the positive regulation relationship between HMGB3 and hTERT. Furthermore, we analyzed the correlation between HMGB3/hTERT expression with clinical variables in these cervical cancer patients, and found that high expression of HMGB3/hTERT was related with comparatively higher TNM stage and poorly differentiation (Table 1). Kaplan-Meier analysis showed that survival probability of patients with both high expression of HMGB3 and hTERT was significantly lower than other groups (Fig. 6e, f). In addition, HMGB3 and hTERT expression was remarkably associated with the 5-year survival of patients (Additional file 2:Table S1). Multivariate Cox proportional hazards model analysis indicated that HMGB3 expression was an independent prognostic risk factor in cervical cancer patients (HR = 23.48, 95% CI: 3.21–171.94, *P* = 0.0019) (Additional file 2:Table S2).

**Table 1:** Correlation analyses of HMGB3/hTERT protein expression in relation to clinicopathologic variables of 119 cervical cancer patients

Characteristic	HMGB3		*P*	hTERT		*P*
	High	Low		High	Low	
Age			0.06			0.07
≤ 56	23	42		39	26	
> 56	38	16		42	12	
Pathology			0.184			0.088
CIN III	8	10		8	10	
CSCC	61	31		65	27	
AUC	3	0		3	0	
mixed	3	2		4	1	
Differentiation			0.027			0.456
High	5	1		5	1	
Middle	10	12		13	8	
Low	53	20		54	19	
T			4.21 × 10^−8^			2.51 × 10^−7^
T1	27	40		31	36	
T2	26	3		27	2	
T3-T4	23	0		23	0	
N			0.0001			0.0004
N0	54	43		59	38	
N1	22	0		22	0	
stage			4.21 × 10^−8^			2.51 × 10^−7^
I	27	40		31	36	
II	26	3		27	2	
III-IV	23	0		23	0	

Taken together, these data indicated that HMGB3 transcriptionally upregulated hTERT expression, and then activated DNA damage repairsignaling pathways, thereby promoting radioresistance and eventual poor outcomes in human cervical cancer (Fig. 6g).

## Discussion

Radioresistance is commonly recognized as the crucial bottleneck for the cure of cervical cancer [6, 28]. Therefore, it is urgent to discover and identify the exact mechanism of radiation resistance and potential targets. In this study, we have identified HMGB3/hTERT signaling axis as a new target for cervical cancer radioresistance.

The HMGB proteins are ubiquitous chromatin-associated DNA binding proteins in mammals, which acts as a DNA chaperone in transcription, replication, recombination and repair [29, 30].An inverse relationship between HMGB expression levels and overall survival has been reported in many cancer types [31–33]. HMGB proteins have 80% homology, so they may have some of the same functions [17]. HMGB1, the most studied member of the HMGB protein family, has pleiotropic roles in cells. HMGB1 could activate the ERK1/2, MAPK, NF-kB and Akt signaling pathways by binding to the receptor for advanced glycation end products (RAGE), leading to the reprogramming of cancer cells. Also, upregulation of HMGB1 was found to contribute to radioresistance in squamous cell carcinoma by promoting chromatin modification and increasing the phosphorylation of CHK1 to activate DNA damage responses (DDR) [33]. HMGB1 is a very important proinflammatory cytokine and can stimulate the immune system to protect against infections. Loss of the pro-inflammatory cytokine functions of HMGB1 may increase the risk of infection and lead to autophagy deficiency, contributing to inflammation [34, 35]. HMGB3 may be more suitable as a target for cancer treatment because of the high expression and non-proinflammatory effect. To date, there is no report on the relationship between HMGB3 and radiotherapy resistance. In our study, we showed that HMGB3 was highly expressed in cervical cancer radioresistant cells and HMGB3 knockdown significantly enhanced the susceptibility of cervical cancer cells to radiation in vivo and in vitro. An inverse relationship between HMGB3 expression and radiotherapy response was also found. Our findings suggest that HMGB3 may be a new target for cervical cancer radiosensitization.

The main mechanism of radiotherapy killing tumor cells is through inducing single and double stranded DNA breaks. The ability of DNA damage detection and repair is closely related to radiosensitivity [36, 37]. Previous studies have showed that HMGB1, HMGB2 depletion impaired radiation-induced DNA damage repair as indicated by increased γ-H2AX foci [38, 39]. Also, HMGB1 and HMGB2 facilitate V(D)J recombination by enhance the affinity of the RAG complexes for DNA and increased the cleavage effectiveness [40, 41]. Inhibition of HMGB3 in cisplatin-resistant ovarian cancer cells resulted in transcriptional downregulation of ATR and CHK1, subsequently attenuating the ATR/CHK1/p-CHK1 DNA damage signalling pathway [19]. In our study, we found that down-regulation of HMGB3 significantly impaired the radiation-induced DNA damage repair and increased cell apoptosis. There are two main pathways responsible for DNA DSB repair: non-homologous end joining (NHEJ) and homologous recombination (HR). The mechanism by which HMGB3 is involved in DNA DSB repair is sti ll uncertain.Telomeres,a specialized DNA/protein structures, can protect chromosomes from end-to-end fusions and from losing coding sequences. Telomeres maintenance is crucial for the stability of chromosome [42]. However, previous studies have revealed overactivity of telomerase is strongly associated with occurrence, metastatic potential and CSC phenotype of malignancy. Telomerase overactivity was also considered as a molecular maker of poor prognosis [43]. hTERT can promote DNA repair and to help cells escape from cell cycle arrest and/or apoptosis, making cancer cells more resistant to chemotherapeutic or radiation therapy [44–46]. Polanska et al. showed that knockout of the HMGB1 gene in mouse embryonic fbroblasts (MEFs) resulted in a decline in telomerase activity and telomere dysfunction, while overexpression of HMGB1 enhanced telomerase activity [47]. Shaobo et al. showed that downregulation of HMGB1 breaks telomere homeostasis by changing the level of telomere binding proteins, such as TPP1 (PTOP), TRF1 and TRF2 and enhances radiosensitivity in human breast cancer cells [48]. Our study showed that HMGB3 could bind to hTERT promoter on the region of − 902 to − 321 and induce the expression of hTERT, leading to radioresistance in cervical cancers. Because of the lack of enzymatic function, HMGB proteins facilitate a stable interactions of other transcription factors with their binding sites in DNA and participate in the regulation process [49]. Whether HMGB3 cooperates or antagonizes with other factors to regulate hTERT needs further study.

The PI3K/Akt signalling cascade plays a crucial role in cell proliferation, growth and apoptosis. It has also been implicated in the cellular response to genotoxic damage. Activated AKT mediates the phosphorylation of hTERT, thereby enhancing telomerase activity [50]. Activated AKT is also known to stimulate c-Myc and to lead to cytoplasmic retention of BRCA-1. BRCA-1 in turn can negatively regulate hTERT and telomerase activation, and presumably it can support the cell survival strategy [51]. The regulatory mechanism of hTERT is complex. Whether HMGB3 cooperates or antagonizes with other factors to regulate hTERT needs further study.

A total of 53 cervical cancer patients receiving radiotherapy enrolled in our study. A significant association was found between HMGB3/hTERT expression and radiosensitivity. Patients with higher expression of HMGB3/hTERT, commonly exhibited worse short-term outcomes in response to radiotherapy. In addition, we further explored the relationship between HMGB3/hTERT expression and clinicopathologic features by means of tumor tissue microarray. The patients who had a high HMGB3 expression similarly displayed a higher percentage of high hTERT expression, hinting the potential regulation of hTERT by HMGB3 in cervical cancer. Moreover, HMGB3/hTERT expression was positively correlated to tumor stage, while negatively corelated with long-term survival. The role of HMGB3 and hTERT in cervical cancer should be better to confirm in a larger cohort.

## Conclusions

In summary, our study has revealed the crucial role of HMGB3 in radioresistance and prognosis in cervical cancer. HMGB3 as a transcription factor, binds to the promoter region of hTERT andactivates transcriptionally hTERT expression, which leads to radiation resistance of cervical cancer.Targeting HMGB3 /hTERT signaling axis may provide an important strategy in cervical cancer treatment.

## Supplementary information

**Additional file 1: Figure S1.** Enhanced DNA damage repair ability in radioresistant cervical cancer cells. **Figure S2.** HMGB3was identified as a new transcriptional factor of hTERT in cervical cancer radioresistant cells. **Figure S3.** Knockdown HMGB3 inhibited DNA damage repair and promote apoptosis in the cervical cancer cells after radiotherapy. **Figure S4.** Knockdown HMGB3 enhanced the chemosensitivity of cervical cancer cells. **Figure S5.** Knockdown of HMGB3 increased the susceptibility to radiotherapy in the xenograft mouse model.

**Additional file 2: Table S1.** Kaplan Meier analysis revealing the correlation between different clinicopathological parameter and 5-year overall survival. **Table S2.** The multivariate Cox proportional hazards model analysis of risk factors showing that TNM stage and HMGB3 expression were independent prognostic risk factors in cervical cancer.

## Data Availability

The authors declare that the data supporting the findings of this study areavailable within the article.

## Abbreviations

HMGB3: High mobility group box3
RR: Radioresistance
RT: Radiotherapy
IR: Irradiation
DSB: DNA double-strand breaks
DDP: Cisplatin
TAX: Paclitaxel
VIN: Vincristine
NHEJ: Non-homologous end joining
HR: Homologous recombination
D_0_ (Gy): Mean lethal dose
D_q_ (Gy): Quasi-threshold dose
N: Extrapolation number
