# Supplementary material for: Targeting HMGB3/hTERT axis for radioresistance in cervical cancer
Source: J Exp Clin Cancer Res. 2020 Nov 13;39:243. doi: 10.1186/s13046-020-01737-1 (PMC7664109; doi:10.1186/s13046-020-01737-1)
Supplement: Supplementary file 1 — Additional file 1: Figure S1. Enhanced DNA damage repair ability in radioresistant cervical cancer cells. Figure S2. HMGB3was identified as a new transcriptional factor of hTERT in cervical cancer radioresistant cells. Figure S3. Knockdown HMGB3 inhibited DNA damage repair and promote apoptosis in the cervical cancer cells after radiotherapy. Figure S4. Knockdown HMGB3 enhanced the chemosensitivity of cervical cancer cells. Figure S5. Knockdown of HMGB3 increased the susceptibility to radiotherapy in the xenograft mouse model. [file 13046_2020_1737_MOESM1_ESM.pdf]

Supplement Figure 1

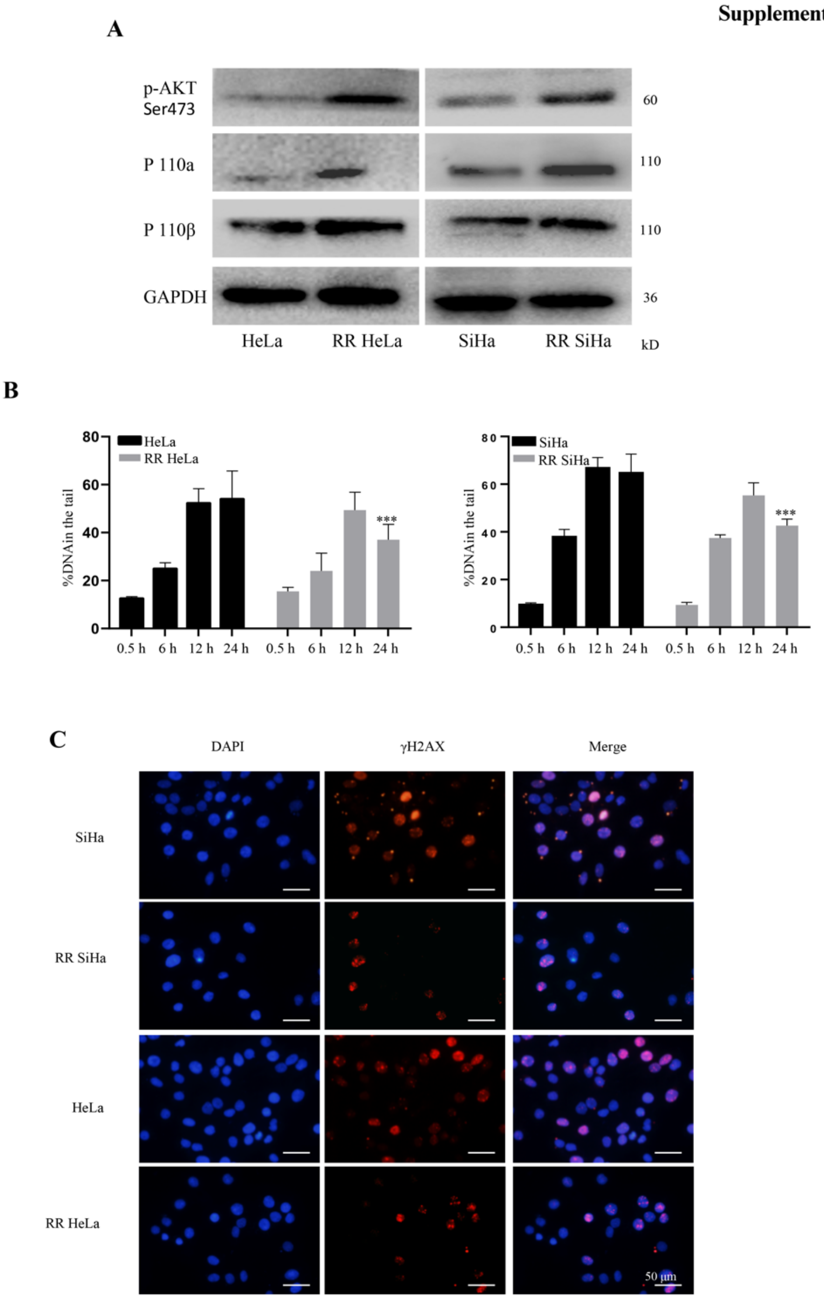

Supplement Figure 1. Enhanced DNA damage repair ability in radioresistant cervical cancer cells

(A) The levels of p110α, p110β, p-AKT in HeLa, SiHa , RR HeLa and RR SiHa were analyzed by Western blot. (B) Comet assay was performed in HeLa, RR HeLa, SiHa and RR SiHa cells at the different time point after exposed to 4 Gy radiation. (C) After 24 hours, immunoblotting of γH2AX in SiHa, HeLa and RR SiHa, RR HeLa cells after treated with 4 Gy radiation.

Supplement Figure 2

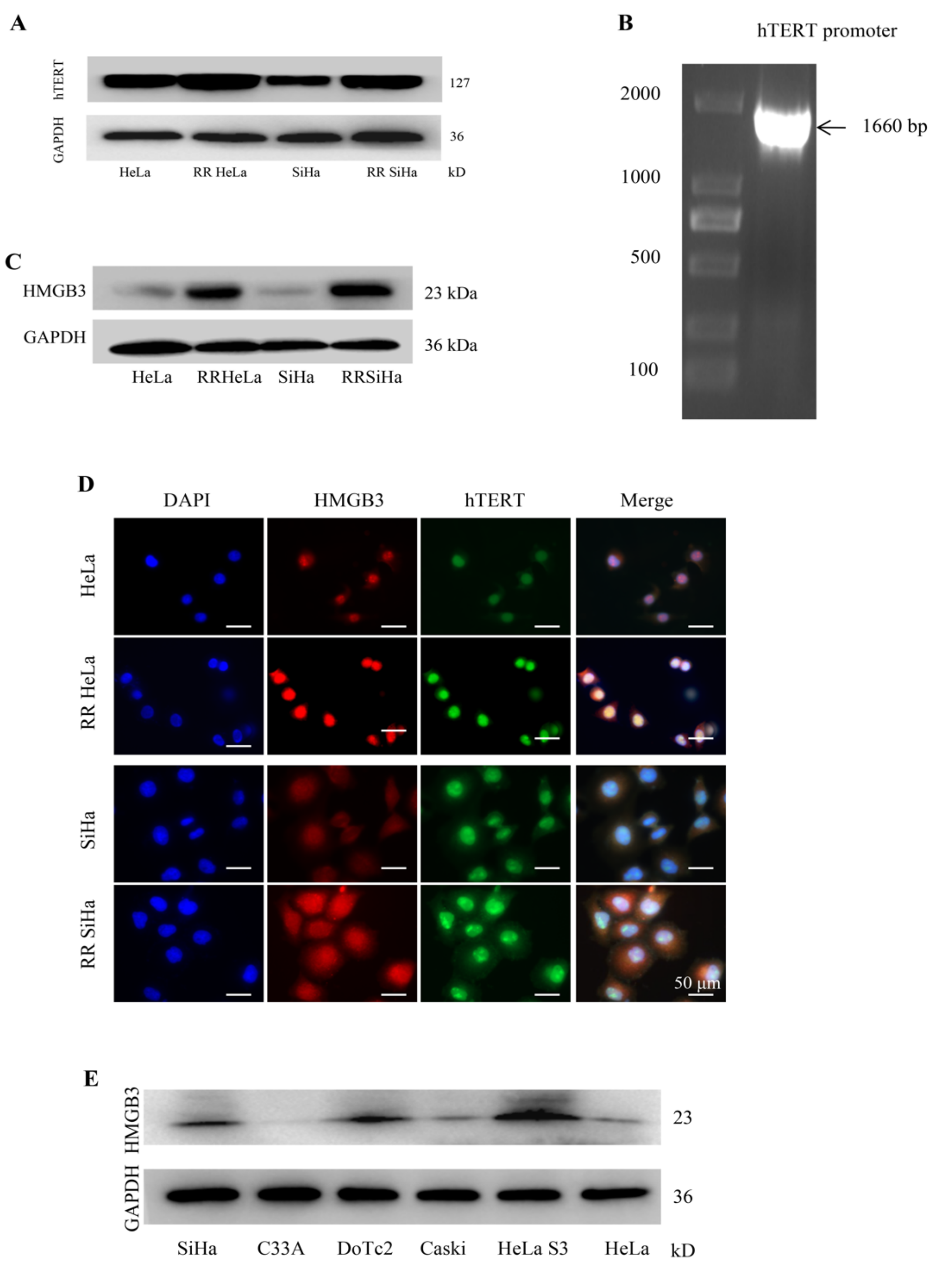

Supplement Figure 2. HMGB3was identified as a new transcriptional factor of hTERT in cervical cancer radioresistant cells

(A) The expression of hTERT in radioresistant and parental cervical cancer cells was checked by western blot. (B) PCR results of hTERT promoter probe with a biotin from -1645 to +15. (C) The expression of HMGB3 in radioresistant and parental cervical cancer cells was checked by western blot. (D) The subcellular localization of HMGB3 and hTERT were detected by confocal laser scanning microscope. (E) HMGB3 expression was detected in six cervical cancer cell lines (SiHa C33A, DoTc2, Caski, HeLa S3, HeLa) by Western blot analysis.

Supplement Figure 3

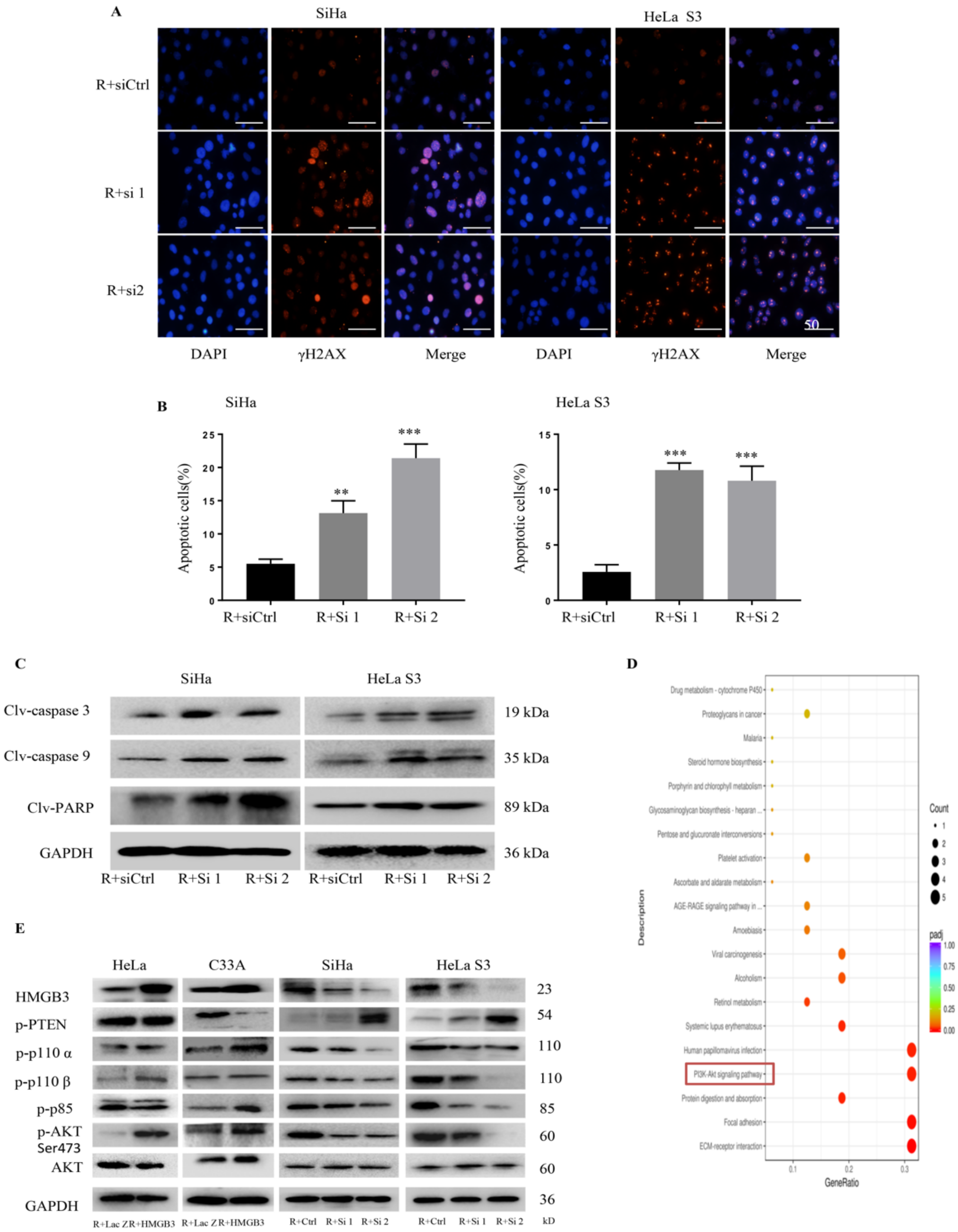

Supplement Figure 3. Knockdown HMGB3 inhibited DNA damage repair and promote apoptosis in the cervical cancer cells after radiotherapy

Cervical cancer cells were transfected HMGB3 specific siRNAs, and then exposed to 4Gy radiation. At 24h, the levels of γ H2AX were detected by immunofluorescence assays (A), and FACS analysis was performed to detect cell apoptosis (B). (C) The expression level of cleaved-caspase-3, cleaved-caspase-9 and cleaved-PARP were detected by western blot. (D) KEGG analysis of the differentially expressed genes. (E) The proteins in PI3K/AKT pathway were analyzed by Western blot 24 h after 4Gy radiation.

Supplement Figure 4

A

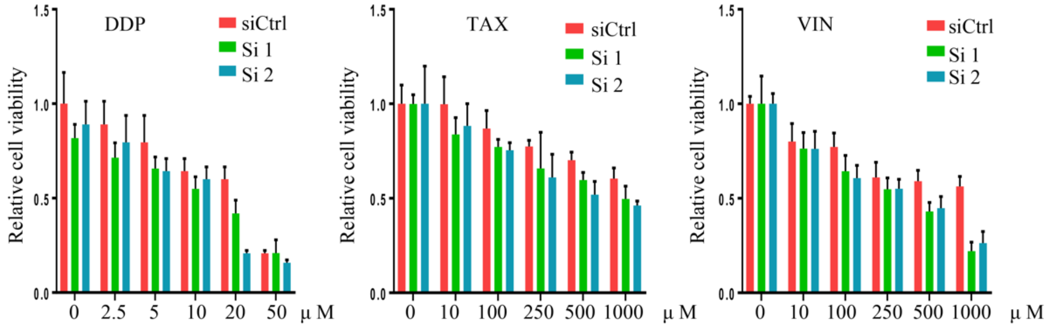

B

	IC50(μ M)		
	DDP	TAX	VIN
siCtrl	20.29	1860.8	2116.29
Si 1	12.73	1188.7	237.9
Si 2	10.22	669.54	238.1

C

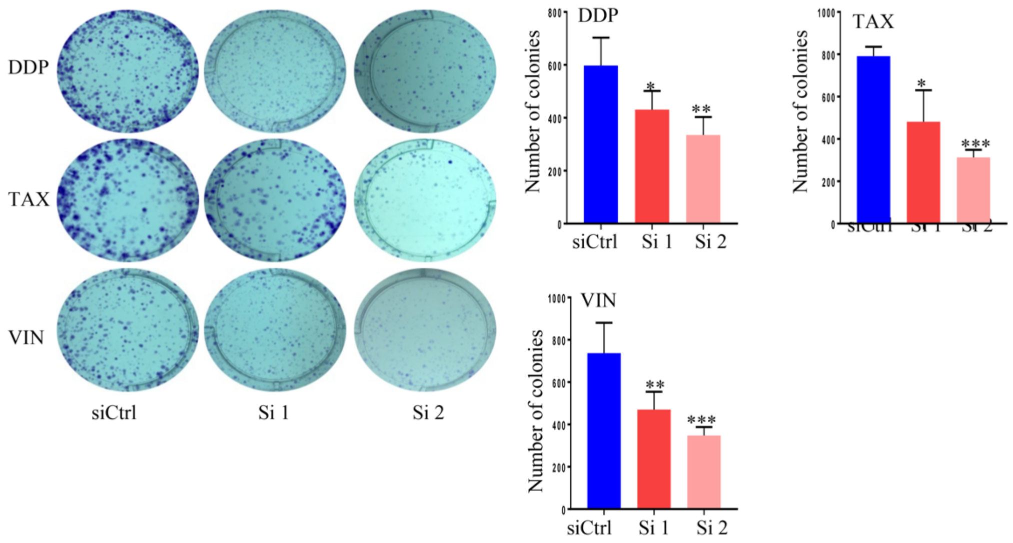

Supplement Figure 4. Knockdown HMGB3 enhanced the chemosensitivity of cervical cancer cells

SiHa was transfected with HMGB3 specific siRNAs, and then treated with different dose of DDP, Vin and TAX. After 48h, MTT assay was used to detected the cell viability (A), the IC50 values of DDP, Vin, TAX was calculated (B). (C) Colony formation was performed for SiHa cells treated with HMGB3 specific siRNAs and DDP (15μ M) or Vin(1 mM) or TAX (500 μM).

Supplement Figure 5

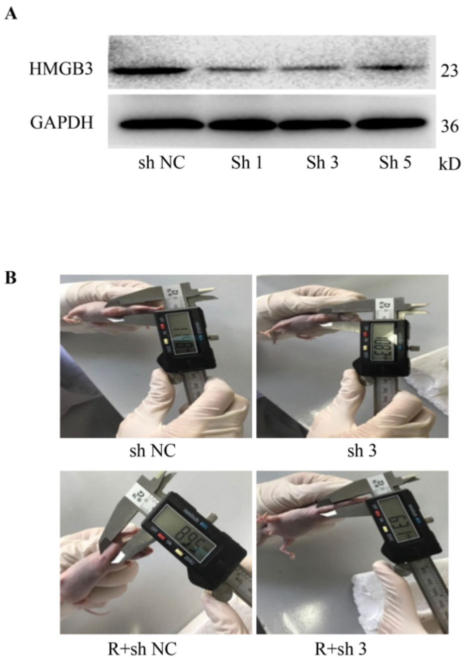

Supplement Figure 5. Knockdown of HMGB3 increased the susceptibility to radiotherapy in the xenograft mouse model

(A) SiHa was infected by lentivirus-HMGB3 shRNA, and the expression of HMGB3 was detected by western blot. (B) Cells were implanted into left armpit of the nude mice.
