# Supplementary material for: Targeting HMGB3/hTERT axis for radioresistance in cervical cancer
Source: J Exp Clin Cancer Res. 2020 Nov 13;39:243. doi: 10.1186/s13046-020-01737-1 (PMC7664109; doi:10.1186/s13046-020-01737-1)
Supplement: Supplementary file 2 — Additional file 2: Table S1. Kaplan Meier analysis revealing the correlation between different clinicopathological parameter and 5-year overall survival. Table S2. The multivariate Cox proportional hazards model analysis of risk factors showing that TNM stage and HMGB3 expression were independent prognostic risk factors in cervical cancer. [file 13046_2020_1737_MOESM2_ESM.docx]

| Characteristic | 5-OS (%) | | P |
| --- | --- | --- | --- |
| Age |  |  | 3.32×10^-8^ |
| ≤56 | 93.8 | |  |
| >56 | 48.1 | |  |
| Differentiation |  |  | 0.256 |
| High | 55 | |  |
| Middle | 76.2 | |  |
| Low | 67.1 | |  |
| T |  |  | 1.27×10^-13^ |
| T1 | 94 | |  |
| T2 | 51.7 | |  |
| T3-T4 | 41 | |  |
| N |  |  | 8.92×10^-8^ |
| N0 | 81.4 | |  |
| N1 | 36.4 | |  |
| stage |  |  | 1.27×10^-13^ |
| I | 94 | |  |
| II | 51.7 | |  |
| III-IV | 41 | |  |
| HMGB3 |  | | 5.16×10^-6^ |
| High | 59.2 | |  |
| Low | 97.7 | |  |
| hTERT |  | | 9.13×10^-6^ |
| High | 60.5 | |  |
| Low | 100 | |  |

Supplement Table 1. Kaplan Meier analysis revealing the correlation between diﬀerent clinicopathological parameter and 5-year overall survival.

| Characteristic | HR | | 95%CI | | P |
| --- | --- | --- | --- | --- | --- |
| stage |  |  |  |  |  |
| I | 1 | | / | | / |
| II | 11.19 | | 3.67-34.13 | | 2.17×10^-5^ |
| III-IV | 4.19 | | 2.41-7.30 | | 4.25×10^-7^ |
| T |  |  |  |  |  |
| T1 | 1 | | / | | / |
| T2 | 11.19 | | 3.67-34.13 | | 2.17×10^-5^ |
| T3-T4 | 4.19 | | 2.41-7.30 | | 4.25×10^-7^ |
| N |  |  |  |  |  |
| N0 | 1 | | / | | / |
| N1 | 5.41 | | 2.71-10.80 | | 1.66×10^-6^ |
| HMGB3 |  |  |  |  |  |
| High | 23.48 | | 3.21-171.94 | | 0.0019 |
| Low | 1 | | / | | / |
| hTERT |  | |  | |  |
| High | Inf | | 0-Inf | | 0.996 |
| Low | 1 | | / | | / |

Supplement Table 2. The multivariate Cox proportional hazards model analysis of risk factors showing that TNM stage and HMGB3 expression were independent prognostic risk factors in cervical cancer.
